# Complete Plastome of Three Korean *Asarum* (Aristolochiaceae): Confirmation Tripartite Structure within Korean *Asarum* and Comparative Analyses

**DOI:** 10.3390/plants10102056

**Published:** 2021-09-29

**Authors:** Mi-Jeong Yoo, Dong-Pil Jin, Hyun-Oh Lee, Chae Eun Lim

**Affiliations:** 1 Department of Biology, Clarkson University, Potsdam, NY 13699, USA; myoo@clarkson.edu; 2 National Institute of Biological Resources, 42 Hwangyeong-ro, Seo-gu, Incheon 22689, Korea; jindp@korea.kr; 3 Phyzen Inc., 13 Seongnam-daero, 331 beon-gil, Bundang-gu, Seongnam-si 13558, Korea; dlgusdh88@phyzen.com

**Keywords:** *Asarum*, plastome, genomic rearrangement, phylogeny, tripartite structure

## Abstract

The genus *Asarum* (Aristolochiaceae) is a well-known resource of medicinal and ornamental plants. However, the taxonomy of Korean *Asarum* is ambiguous due to their considerable morphological variations. Previously, a unique plastome structure has been reported from this genus. Therefore, we investigated the structural change in the plastomes within three Korean *Asarum* species and inferred their phylogenetic relationships. The plastome sizes of *Asarum* species assembled here range from 190,168 to 193,356 bp, which are longer than a typical plastome size (160 kb). This is due to the incorporation and duplication of the small single copy into the inverted repeat, which resulted in a unique tripartite structure. We first verified this unique structure using the Illumina Miseq and Oxford Nanopore MinION platforms. We also investigated the phylogeny of 26 Aristolochiaceae species based on 79 plastid protein-coding genes, which supports the monophyly of Korean *Asarum* species. Although the 79 plastid protein-coding gene data set showed some limitations in supporting the previous classification, it exhibits its effectiveness in delineating some sections and species. Thus, it can serve as an effective tool for resolving species-level phylogeny in Aristolochiaceae. Last, we evaluated variable sites and simple sequence repeats in the plastome as potential molecular markers for species delimitation.

## 1. Introduction

The genus *Asarum* L. (Aristolochiaceae), which consists of about 100 species, is mainly distributed in temperate regions of the Northern Hemisphere [1,2,3,4,5]. Most of the species (~80 species) occur in East Asia, including China, Japan, and Korea, while approximately 15 species and a single species are distributed in North America and Europe, respectively [1,6,7]. *Asarum* species are well known as oriental folk medicinal herbs and have horticultural potential as well [8,9,10,11,12,13]. In particular, the dried roots and rhizomes of *Asarum* species (common name “Seshin” in Korea, “Xixin” in China, “Saishin” in Japan, and “Wild ginger” in England) are widely used as a drug in traditional medicinal practices worldwide [14,15]. *Asarum sieboldii* Miq. and *A. heterotropoides* F. Schmidt are valued as remedies for aphthous stomatitis, toothache, and gingivitis in Korea and China [10,16,17]. Taxonomically, these medicinal herbs are placed in the section *Asiasarum* in subgenus *Heterotropa* of genus *Asarum* [18].

The section *Asiasarum*, distributed in northeastern Asia (China, Japan, Korea, and eastern Russia), is defined by perennial rhizome, deciduous leaves, connate style at the base, longitudinally ridged inner surface of calyx tube, and half-inferior to superior ovary [19,20,21,22]. In Korea, these taxa are highly variable in morphology, resulting in taxonomic confusion in delimiting species boundaries and determining relationships [18]. The potential interspecific hybridization/introgression could also contribute to this controversy [23,24]. Therefore, variable numbers of *Asarum* taxa from two to 19 species have been described in Korea, but some scientific names seem to be incorrect [5,18,25,26,27]. To establish taxonomic delimitation and phylogenetic relationships among the *Asarum* taxa, including *Asiasarum*, several molecular studies have been conducted. However, these studies employed partial DNA regions, for example, the *trnL*–*trnT* intergenic region, *rpoB*–*trnC*-*GCA*, and *rps16*–*trnK* regions of chloroplast DNA, and the internal transcribed spacer (ITS) region of nuclear ribosomal DNA [28,29,30,31]. However, high sequence similarity in closely related taxa and/or insufficient taxon sampling is an impediment in taxonomic delimitation and in resolving phylogenetic relationships.

Chloroplasts are uniparentally inherited organelles in plant cells. They play important roles in many cellular functions, including photosynthesis, carbon fixation, and stress responses. In angiosperms, the plastome has a well-conserved quadripartite structure composed of two copies of inverted repeats (IR), one large single copy (LSC), and one small single copy (SSC) [32]. In general, the plastomes of most plants are circular DNA molecules ranging in size from 120 to 160 kilobases (kb). However, several rearrangements in the plastomes have been reported, for example, extensive rearrangement of gene order in Fabaceae [33] and *Passiflora* [34], extensive or multiple gene loss in *Geosiris* (Iridaceae) [35], Orobanchaceae [36,37], and Orchidaceae [38], and drastic changes in the IR regions in Arecaceae, Geraniaceae, and Schisandraceae [39,40,41]. Syntenically disrupted and otherwise divergent plastomes have been reported in some American *Asarum* species, which were suggested as contigs of the sequence, but not a completely circularized map [42]. The structural change was supported based on the complete plastome map of *A*. *sieboldii*, a member of the section *Asiasarum* [43]. However, it remains unclear whether changes in the IRs boundary would be present throughout the section. These rearrangements in the plastome might provide important evidence for inferring the phylogenetic relationship and evolutionary history of their lineage. Additionally, the variable regions of the plastome have been developed as DNA barcode systems to identify each taxon. Owing to the improvements in sequencing technologies, the whole plastome sequence is often used as a super-barcode for the identification of plants these days [44].

In this study, we investigated the plastomes of four Korean *Asarum* species (all members of section *Asiasarum*) suggested by Oh [5] to generate robust data for resolving taxonomic/phylogenetic problems. We sequenced and assembled the whole plastomes of three *Asarum* species (*A. heterotropoides*, *A*. *misandrum* B. U. Oh and J. G. Kim, and *A*. *maculatum* Nakai) and one related species (*Aristolochia contorta* Bunge). Additionally, to confirm the rearrangement events and structural changes in the plastome of Korean *Asarum* species, we used long reads produced by Oxford Nanopore Technology (ONT) MinION together with short reads generated by Illumina MiSeq for *A*. *maculatum*. Moreover, a comparative analysis of the plastomes was conducted using previously published data to establish a molecular basis for the development of novel DNA barcode markers and to infer the phylogenetic relationships of Aristolochiaceae.

## 2. Results

### 2.1. Plastome Features and Gene Content

**Figure 1:**
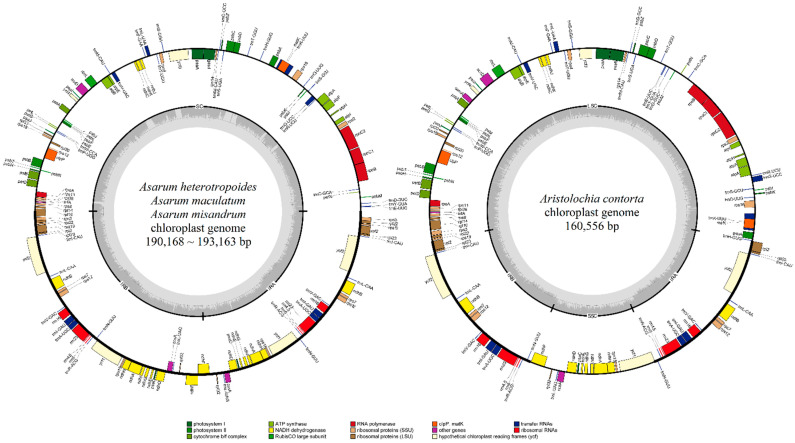
The plastome maps of three Korean *Asarum* (**left**) and one *Aristolochia* (**right**) species. Genes are represented in different colors according to their functions. Transcriptional directions are denoted on the inside (clockwise) and the outside (counterclockwise) of the circle.

A total of four complete plastomes (three *Asarum* and one *Aristolochia* species) were newly sequenced and successfully assembled using high-quality Illumina MiSeq and ONT MinION data. In detail, we acquired 1.35 to 1.70 Gbp of Illumina MiSeq short reads per species and 2.22 Gbp of ONT MinION long reads for one species, *A. maculatum*. For Illumina MiSeq data, the number of reads after quality trimming (>q20) was about 4,624,218 to 6,918,313, and the average coverage for the plastome ranged from 549 to 2019 (Table S1). For ONT MinION data, the number of reads after quality trimming (> q7) was 523,864, and the average coverage for the plastome was 291 (Table S1). The complete plastome size of the three Korean *Asarum* species (*A. heterotropoides*, *A. misandrum*, and *A. maculatum*) ranged from 190,168 to 193,163 bp, and the GC ratio ranged from 36.22% to 36.78% (Figure 1; Table 1). 

These plastomes had tripartite structures consisting of a single copy (SC) region ranging from 93,336 to 96,357 bp and a pair of IRs ranging from 48,402 to 48,416 bp. The complete plastome of *Aristolochia contorta* was 160,556 bp in length and was smaller than that of *Asarum* species. The GC ratio of *Aristolochia contorta* was 38.49%, being slightly higher than those of *Asarum* species. The genome structure of *Aristolochia contorta* had a typical quadripartite structure unlike those of *Asarum* species, with LSC (89,756 bp), SSC (19,882 bp), and a pair of IRs (25,459 bp) (Figure 1; Table 1).

The plastomes of four species in Aristolochiaceae contained 112–113 genes, consisting of 78–79 protein-coding, 30 tRNA, and 4 rRNA genes (Table 2). The gene contents of *Asarum* and *Aristolochia* were largely identical, except for the duplicated genes. In three *Asarum* species, 33 genes were duplicated in the IR region: *ccsA*, *ndhA*, *ndhB*, *ndhD*, *ndhE*, *ndhF*, *ndhG*, *ndhH*, *ndhI*, *psaC*, *rpl2*, *rpl22*, *rpl23*, *rpl32*, *rps3*, *rps7*, *rps12*, *rps15*, *rps19*, *rrn4.5*, *rrn5*, *rrn16*, *rrn23*, *trnA-UGC*, *trnI-CAU*, *trnI-GAU*, *trnL-CAA*, *trnL-UAG*, *trnN-GUU*, *trnR-ACG*, *trnV-GAC*, *ycf1*, and *ycf2*. In contrast, only 17 genes (*ndhB*, *rpl2*, *rpl23*, *rps7*, *rps12*, *rrn4.5*, *rrn5*, *rrn16*, *rrn23*, *trnA-UGC*, *trnI-CAU*, *trnI-GAU*, *trnL-CAA*, *trnN-GUU*, *trnR-ACG*, *trnV-GAC*, and *ycf2*) were duplicated in the IR region in *Aristolochia contorta*. This difference between genera was caused by the IR expansion. Among 16 genes that were only duplicated in *Asarum*, three genes (*rpl22*, *rps3*, and *rps19*) were duplicated due to IR expansion toward LSC, whereas 13 genes (*ccsA*, *ndhA*, *ndhD*, *ndhE*, *ndhF*, *ndhG*, *ndhH*, *ndhI, psaC*, *trnL-UAG*, *rpl32*, *rps15*, and *ycf1*) were duplicated because the entire SSC was integrated into the IR region (Figure 1, Table 2). In the comparison of genes, *cemA* was pseudogenized in *A*. *misandrum* due to insertion of A-polymer at the 5’-portion, while the genes of other *Asarum* species were intact.

### 2.2. Comparative Plastome Structure and Polymorphism

**Figure 2:**
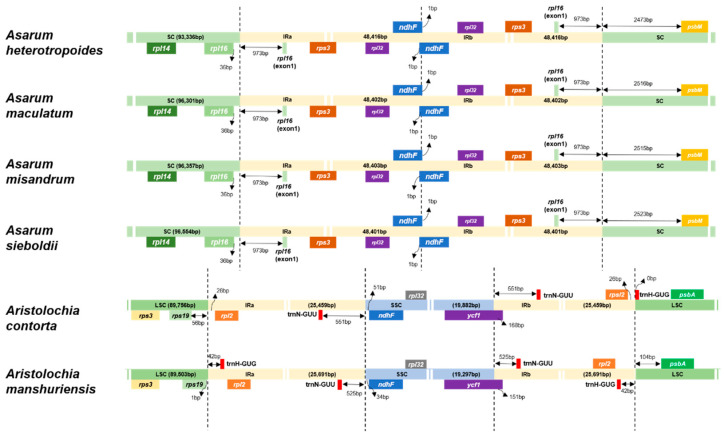
Comparison of the boundaries between regions within the plastome. Each region is represented as a bar: Large single copy (LSC) (including SC), pale-green; inverted repeat (IR), pale-yellow; small single copy (SSC), pale-blue. The genes on the bar are transcribed (from left to right), whereas the genes under the bar are transcribed (from right to left).

To understand synteny (gene order) and polymorphism in the plastomes in Aristolochiaceae occurring in Korea, we compared the SC/IR boundaries in six Korean Aristolochiaceae species, including the plastome of *A*. *sieboldii* of section *Asiasarum* previously published [43]. The comparative analysis revealed that the plastomes of *Asarum* species were different from that of *Aristolochia* in terms of structures (Figure 1 and Figure 2). A disruption in the SSC region was found in the plastomes of *Asarum* compared to the plastome of *Aristolochia* (Figure 1). This involved the incorporation of the entire SSC into the IRs, implying that the plastome of *Asarum* species is functionally bipartite. In contrast, the plastome of *Aristolochia* species has a quadripartite structure typical of angiosperms (Figure 1). We compared the duplicated genes between *Asarum* and *Aristolochia* species. A total of 16 genes, detected on the SSC of *Aristolochia*, were duplicated in the IRs of *Asarum* (see above). For *ndhF*, this is located on the SSC/IRB boundary in *Aristolochia* species, but extends on the boundary of two IRs, with 1 bp of the 3′ portion in each other IR, in *Asarum* (Figure 2). The *ycf1* gene, placed at the SSC/IRA boundary in *Aristolochia*, is nested interior to the IR in *Asarum* (Figure 1). In the SC of *Asarum*, the region from *trnH-GUG* to *trnE-UUC* is inversed, when compared with the LSC of *Aristolochia* (Figure 1). The comparison of the plastomes from the same *Asarum* species also showed these patterns. When two plastome sequences of *A. sieboldii* [MG551543 (193,356 bp) vs. MW034667 (167,293 bp)] and *A. heterotropoides* [MN132860 (190,168 bp) vs. MK577409 (159,944 bp)] were compared, two distinct variations were identified. The first one was the reversed orientation of some LSC area which corresponds to the region from *trnH-GUG* to *trnE-UUC* (blue arrow in Figure S2), and the second one was the incorporation of SSC into the IR regions (red and orange arrows in Figure S2). This synteny and rearrangements in the plastome of *Asarum* were verified using the combination of two sequencing datasets (ONT MinION and Illumina MiSeq data) from the identical sample of *A*. *maculatum*. All boundaries in the plastome of *Asarum* were well assembled by those reads (Figure S1).

**Figure 3:**
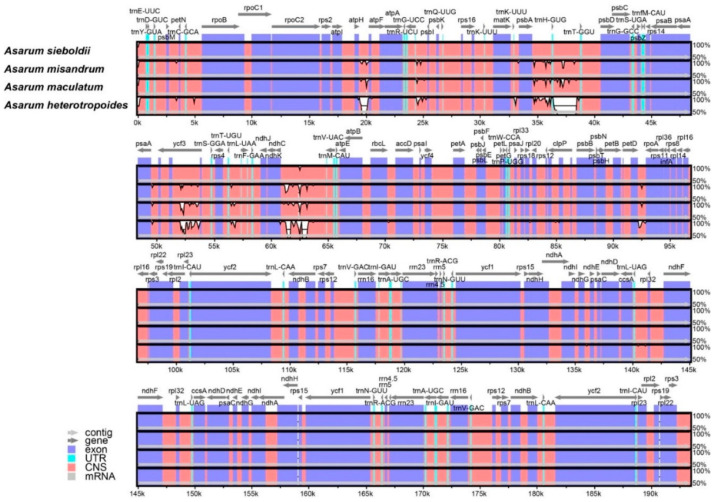
Comparison of the plastome structure of *Asarum* species using the mVISTA program. Sequence regions are colored according to their types. Transcriptional direction of the gene is represented by the direction of the arrow. The percentage on the right side indicates sequence identity among plastomes (50% to 100%). The number under the slot indicates the position in the genome. CNS = non-coding sequences, UTR = untranslated region.

**Figure 4:**
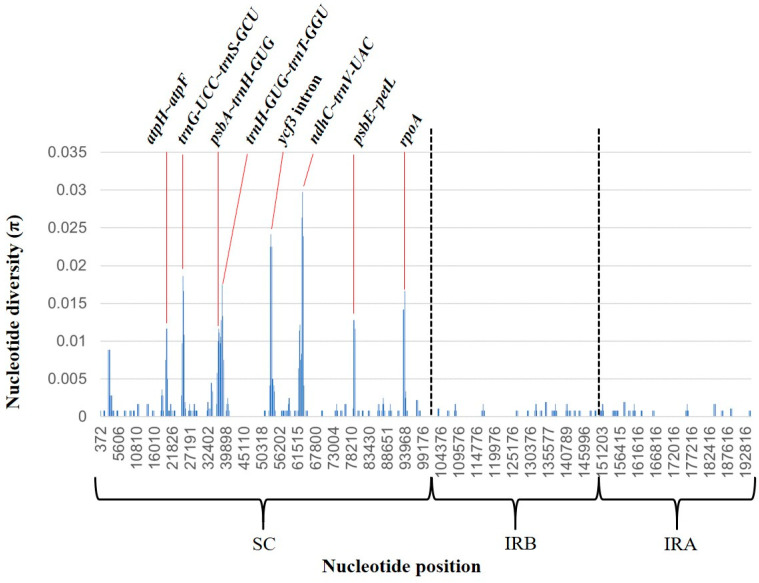
Nucleotide diversity (π) in the chloroplast genome of four Korean *Asarum* species. *x*-axis, nucleotide position; *y*-axis, nucleotide diversity of each region. The regions that show a high value (>0.01) are marked by a red line.

Comparison of the plastome polymorphism in *Asarum* species revealed that the plastome sequences are fairly conserved across the four species except for a few regions with variations (Figure 3). The sequences of exons and untranslated regions are nearly identical throughout the four *Asarum* species, whereas most of the detected variations were found in the non-coding sequence (Figure 3). Within *Asarum*, the largest size of the IR was seen in *A*. *heterotropoides* despite its smallest overall plastome size (Table 1), resulting from nucleotide indel in the non-coding sequence. Distance from the boundary to genes was compared within these species. The distances between exon 1 of *rpl16* and the SC/IR boundary are identical within Korean *Asarum* (973 bp), but the location of *psbM* with respect to the SC/IRA boundary varies across species. Among the selected taxa, the location of the gene in *A*. *sieboldii* is the farthest from the boundary (2523 bp), whereas in *A*. *heterotropoides*, it is the closest from the boundary (2473 bp) (Figure 2). We also examined nucleotide polymorphism (π, nucleotide diversity) among the four Korean *Asarum* species (Figure 4). The average π was 0.0009, and the value of π ranged from 0 to 0.0297. Overall, nucleotide sequences of the IRs are more conserved (average π = 0.0003) than those of SC (average π = 0.0016). The most highly variable regions (π > 0.020) include one protein-coding gene (*rpoA*: π = 0.015) and seven intergenic spacers (IGS) (*ycf3* intron: π = 0.023, *ndhC*–*trnV*-*UAC*: π = 0.020, *trnG*-*UCC*–*trnS*-*GCU*: π = 0.015, *trnH*-*GUG*–*trnT*-*GGU*: π = 0.014, *psbE*–*petL*: π = 0.012, *atpF*–*atpH*: π = 0.011, *psbA*–*trnH-GUG*: π = 0.010).

### 2.3. Repeat Sequences in the Plastome of Asarum Species

The simple sequence repeats (SSRs) in the plastomes of the four Korean *Asarum* species were analyzed. The total number of SSRs found in the plastome of these species ranges from 953 to 1039 (Table S2). The mono-, di-, tri-, tetra-, penta-, and hexa-nucleotide SSRs were detected among them, and the number of each SSR in the plastome varies across the four *Asarum* species (Table 3 and Table S2). In all the species, the most abundant type of SSR is the penta-nucleotide repeat (494 to 547 in number), whereas the least abundant type is the tetra-nucleotide repeat (6 to 7 in number). Except for the mononucleotide SSR with G or C base located in the IGS of *trnQ-UUG–rps16*, all mono-repeats are composed of A or T base in all the four species. All dinucleotide SSRs are AT or TA base. We further investigated the long sequence repeats (LSRs) shared by the Korean *Asarum* species (Table S3). A total of 49 LSRs were identified, consisting of an average of 7 palindromic, 20 forward, 16 reverse, and 4 complement repeats. Of these, the longest unit size (120 bp) of the repeat was found in *atpH–atpF* of A. sieboldii, whereas the shortest unit size (42 bp) of the repeat was detected in *trnH-GUG–trnT-GGU* of A. maculatum. Most of the repeats (86–92%) are less than 90 bp and nearly a quarter of the repeats (about 16–24%) are situated in or at the border of genic regions. Among the palindromic repeats within the coding region, there is one palindromic repeat on ycf3 of *A. maculatum*, and no palindromic repeat on *ycf3* of the other three *Asarum* species.

### 2.4. Phylogenetic Inference

**Figure 5:**
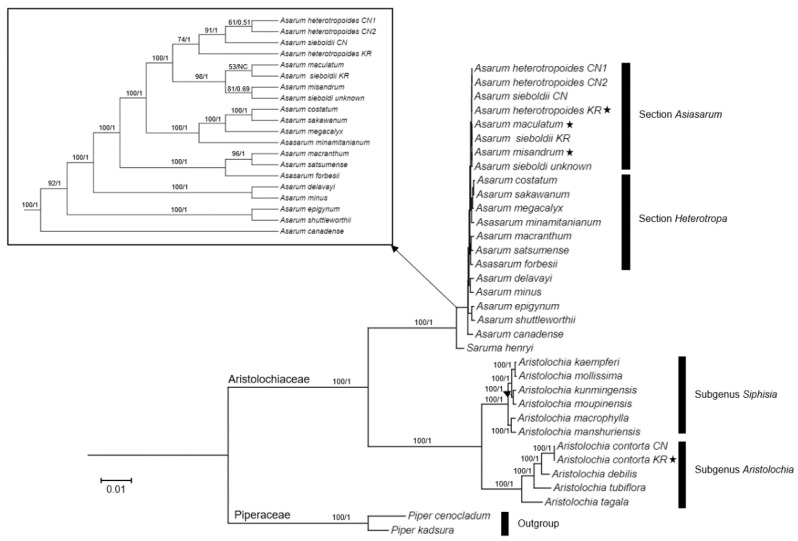
Maximum likelihood (ML) analysis of Aristolochiaceae based on 79 plastid protein-coding genes. The first and second number on each node indicate the bootstrap value of ML and posterior probability of Bayesian inference, respectively. The NC means “Not calculated”. Newly sequenced individuals in this study are marked with a star. A cladogram of *Asarum* species was presented in the box to show their relationships. CN and KR next to taxon name indicate Chinese and Korean accession, respectively.

The Maximum likelihood (ML) tree was constructed to explore the phylogenetic relationships among 26 species of Aristolochiaceae using 79 protein-coding gene sequences. This analysis included the four Korean *Asarum* species (newly sequenced species and previously reported *A*. *sieboldii*). The overall topology of the phylogeny computed from ML and Bayesian inference (BI) analyses were identical (BI tree not shown). In the phylogeny, all the *Asarum* species examined here were strongly resolved as a monophyletic group (bootstrap value [BV] of ML/posterior probability [PP] of BI = 100/1) in Aristolochiaceae (Figure 5). *Asarum canadense* L., a member of section *Asarum* of subgenus *Asarum*, was sister to all the remaining *Asarum* species, agreeing with the previous analysis [42]. However, subgeneric classification was not supported by our analysis. For example, *A. epigynum* Hayata belongs to subgenus *Geotaenium*, but it formed a clade with *A. shuttleworthii* Britten and Baker fil., a member of section *Hexastylis* of subgenus *Heterotropa.* All the remaining *Asarum* species are members of subgenus *Heterotropa*, but its monophyly was not supported since *A. shuttleworthii* was not included (Figure 5). Members of section *Heterotropa* formed two clades, while members of section *Asiasarum* formed a monophyletic clade supported by high BV/PP (100/1) (Figure 5). In a clade of section *Asiasarum*, two clades were formed. One clade includes three Korean *Asarum* species (*A*. *maculatum*, *A*. *misandrum*, and *A. sieboldii*) and *A. sieboldii* from unknown locality [42], while another one contains Chinese accessions of *A. sieboldii* and *A. heterotropoides* and Korean *A. heterotropoides* although the latter one received weak support (BV = 74) (Figure 5). *Saruma henryi* Oliv., a monotypic species in this genus, was positioned as the sister to the genus *Asarum* (Figure 5). In addition, 10 species of *Aristolochia* formed another strongly supported monophyletic group and were divided into two subclades (1) subgenus *Siphisia* (BV/PP = 100/1) and (2) subgenus *Aristolochia* (BV/PP = 100/1) (Figure 5). 

Additional phylogenetic analysis with reassembled plastome sequences resolved similar relationships among *Asarum* species, except the clustering of *A. maculatum* and *A. misandrum* (Figure S3). 

## 3. Discussion

### 3.1. Structural Changes in the Plastome of Korean Asarum Species

In this study, we demonstrate the tripartite plastome structure in Korean *Asarum* using a hybrid strategy of ONT MinION in combination with Illumina MiSeq. Prior to sequencing the genome with the ONT MinION platform, we tried to validate the IRA/IRB boundary with a conventional PCR, but the region was not amplified. It was inferred that the sequence of the IRA/IRB boundary region formed a hairpin or cruciform structure which made the amplification of this region by PCR difficult. Therefore, we verified the tripartite structure identified in all Korean *Asarum* species using ONT read mapping (Figure S1). A recently developed hybrid pipeline including a base error correction tool on the ONT long reads along with Illumina short reads showed higher accuracy than the assembly done with short reads only [45,46,47,48]. Our sequencing results showed that the structural variations were caused by the incorporation and duplication of the SSC into the IR regions. In addition, the complete plastomes of three *Asarum* and one *Aristolochia* species were sequenced (Figure 1; Table 1), and the organization of genes was characterized (Table 2). Structural variations across taxa in the family, found in this study, might be applicable for phylogenetic studies.

Angiosperm plastomes generally have very little variation at the structural and genic levels, including length, GC ratio, gene order (synteny), and gene content [49,50]. However, the analysis of complete plastomes of *Asarum* in this study revealed that the structure is syntenically different from those of other plastomes reported from the Magnoliids, including their closely related genera, such as *Aristolochia* and *Saruma* in Aristolochiaceae (Figure 1). This result supports previous genomic studies [42,43]. The plastome structure of the Korean *Asarum* section *Asiasarum* is functionally bipartite because of the incorporation of the SSC into the IRs (Figure 1). For such incorporation of the SSC into the IRs, palindromic sequences in the 3′ portion of *ndhF*, was inferred as a factor for the structural change [42]. Palindromic sequences could take the shape of a cruciform and are associated with chromosome break that leads to rearrangement in genomes [51]. However, it seems that such structural changes occur independently according to section [42]. Three species belonging to each section of subgenus *Heterotropa*, *A*. *minus* F. Maek. (section *Hexastylis*), *A*. *megacalyx* (F. Maek.) T. Sugaw. (section *Heterotropa*), and *A*. *delavayi* Franch. (section *Longistylis*), had a functionally tripartite structure with expanded inverted repeats, while *A*. *canadense* (section *Asarum* of subgenus *Asarum*) and *A*. *sieboldii* (section *Asiasarum*) were characterized by tripartite structures (= functionally bipartite) [42]. Additionally, there were differences among species in the placement of *ndhF*, which is placed on the IRA/IRB boundary. In the case of *A*. *canadense*, 12 bp of the 3′ portion of *ndhF* expanded in another IR, whereas only 1 bp of the gene intruded the IR in *A*. *sieboldii* [42]. Although Lim et al. [43] have reported such structural changes around *ndhF* in *A*. *sieboldii*, it is not clear whether these structural changes are shared within the section. In this regard, our observation that only 1 bp of the 3′ portion of *ndhF* intruded the IR was conserved in Korean *Asarum* is notable (Figure 2); this could be an important genomic event that distinguishes this section from others. We confirmed this feature by reassembling short reads from Chinese accessions of *A. sieboldii* and *A. heterotropoides* and three species of section *Heterotropa*: *A. costatum* (F. Maek.) T. Sugaw., *A. minamitanianum* Hatus., and *A. sakawanum* Makino. The IRA/IRB by *ndhF* intrusion was only observed in *A. sieboldii* and *A. heterotrpoides*, but not in members of section *Heterotropa* (Figure S4). Therefore, it seems that this functionally bipartite structure could be unique to section *Asiasarum*. 

In comparison with the *Aristolochia* plastome, there is another positional shift on the SC (=LSC in *Aristolochia*)/IR boundaries of the plastomes within Korean *Asarum*; this is the incorporation of a part of the SC region (*rps3*, *rpl22*, *rps19*, and partial *rpl16*) into the IRs (Figure 1). This implies that the SC/IRs boundary is also unstable in Korean *Asarum*. In addition, we rechecked the inversion of a large portion including all genes between *trnT*-*GUU* and *trnH*-*GUG* in the SC region in the plastomes in this section (Figure 1). A mechanism for the rearrangement of the SC in *Asarum* was described, and 12 bp inverted repeats with low complexity (AATATAAATAAT) flanking *trnT*-*GUU* and *trnH*-*GUG* were suggested as factors for the rearrangement [40]. Genomic rearrangement by the IRs has been reported across many lineages, such as *Monsonia* (Geraniaceae), *Trachelium* (Campanulaceae), and the tribe Desmodieae (Fabaceae) [52,53,54]. The discussion on such events could help understand the evolution of the plastome in Korean *Asarum*.

### 3.2. Inferring the Molecular Phylogeny of Aristolochiaceae

The genus *Asarum* is well known for taxonomic difficulty due to morphological similarities, high variability of diagnostic characteristics, and natural hybridization [6,7,22,23,24,28,30]. In previous studies, the taxonomic assessment of *Asarum* has been conducted based on flower, leaf, cataphyll, and fruit morphology with a few molecular markers, which may in part complicate the problems [1,18,23,24,30,31]. The whole plastome or plastid protein-coding genes have shown considerable values for reconstructing phylogenetic relationships among the complex taxa at various taxonomic levels during the past decade [55,56,57]. Therefore, we utilized 79 plastid protein-coding gene sequences from 26 taxa in Aristolochiaceae to infer phylogenetic relationships among the four Korean *Asarum* species and the related species (Figure 5). The phylogenetic relationships among 26 taxa were partially congruent with the previously reported relationships for Aristolochiaceae [7,58,59]. Our data showed that members of the family were clustered into two clades, *Aristolochia* and *Asarum*/*Saruma*. However, within the genus *Asarum*, classification at subgeneric and sectional levels was not supported. For example, *A. epigynum*, a member of section *Geotaenium* of subgenus *Geotaenium*, formed a clade with *A. shuttleworthii* (section *Heterostylis* of genus *Heterotropa*) (Figure 5). Since we included single species for most of sections, the utility of the plastome in delineating classification of *Asarum* should be tested with more samples, as well as nuclear data. Nonetheless, members of section *Heterotropa* failed to form a monophyletic group. Since section *Heterotropa* includes many species (about 80 species), its monophyly needs to be investigated with thorough sampling of taxa and nuclear genomic regions. Interestingly, two clades formed by species of section Heterotropa correspond to their geographic distributions: one clade is composed of *A. costatum*, *A. sakwanum*, and *A. megacalyx* which occur in Japan, while the remaining species distribute in southern China and Taiwan. The section *Asiasarum* formed a monophyletic group which is sister to the Japanese *Heterotropa* clade in the ML phylogeny (Figure 5). Although the branch length was very short, this result is congruent with the previous sister relationships between section *Asiasarum* and *Heterotropa* based on nuclear and plastid data sets [30]. In the clade of section *Asiasarum*, Korean species did not form a clade due to low sequence divergence. However, three accessions of *A. heterotropoides* were clustered together with Chinese accessions of *A. sieboldii* collected from eastern China close to North Korea (Figure 5), indicating either misidentification of *A. sieboldii* or introgression of the plastome of *A. heterotropoides* into *A. sieboldii*. Korean accession of *A. sieboldii* was included in the clade of other Korean *Asarum* species along with another *A. sieboldii* which of locality is unknown [42]. This result reflects the taxonomic confusion in *A. sieboldii* which has many synonyms. Further analyses with more accessions could resolve the entity of *A. sieboldii* occurring in the Korean peninsula. Two endemic species of *A*. *maculatum* and *A*. *misandrum* are closely related based on the nearly complete plastome sequences (Figure S3). These two species exhibit morphological differentiation, for example, *A. misandrum* has recurved calyx lobes and a glabrous abaxial surface, while *A. maculatum* has calyx, which is not curved, and whitish blotches on the adaxial leaf surface [5]. Thus, this result might suggest that these two endemic species have shared a maternal parent sometime in their evolutionary history. However, ITS phylogeny showed that *A*. *misandrum* is separated from the remaining Korean *Asarum* species [31], thus, more samples with nuclear data should be investigated further to resolve phylogenetic relationships among Korean *Asarum* species. Although 79 plastid protein-coding genes showed some limitations in supporting the previous classification, it exhibits its effectiveness in delineating some sections and species, therefore, it can serve as an effective tool for resolving species-level phylogeny in Aristolochiaceae.

### 3.3. Molecular Identification

The precise identification of *Asarum* species is essential to use them as a medicinal resource, but it is often restricted by considerably morphological variation within species. Although DNA barcoding was implemented based on universal barcode markers, such as ITS, *rbcL*, and *matK*, for accurate usage of Korean *Asarum* as a medicinal herb, the discriminatory power at the species level was very low [60]. In such a case, the discovery of novel barcodes with high discriminatory power or the use of the whole plastome as a super-barcode seems to be necessary. Therefore, we identified eight hyper-variable sites located in the SC region through comparison of taxa (Figure 4); these comprised, one from *rpoA*, six from the IGS (*atpF*–*atpH*, *psbA*–*trnH*-*GUG*, *ndhC*–*trnV*-*UAC*, *psbE*–*petL*, *trnG*-*UCC*–*trnS*-*GCU*, and *trnH*-*GUG*–*trnT*-*GGU*), and one from the intron of *ycf3*. None of these regions were employed in previous phylogenetic studies [1,30,31,59]. Of these variable regions, *trnG-UCC*–*trnS-GCU* and *ndhC*–*trnV*-*UAC* are also highly variable in the genus *Aristolochia*. Given this fact, these regions could be estimated as candidates for DNA barcodes on the genus *Asarum* as well as on the family Aristolochiaceae. Various SSR regions of four Korean *Asarum* species were detected (Table 3), and some of these loci could be used as markers to infer the historical migration route and genetic diversity/structure within a species. Numerous population genetics studies employed plastid SSR as well as nuclear SSR [61,62,63]. Our SSR data show notable differences in type (tetra-nucleotide) between *A*. *heterotropoides* and remaining species (Table 3 and Table S2), supporting its isolation from the remaining Korean *Asarum* species in the ML tree (Figure 5). Palindromic repeat was only observed in *A*. *maculatum* (Table S1). These sequence variations in the CP genome are potential markers for identification of species.

## 4. Materials and Methods

### 4.1. Sampling, DNA Library Preparation, and Sequencing

Fresh young leaves of three *Asarum* species (*A*. *misandrum*, *A*. *maculatum*, and *A*. *heterotropoides*) and *Aristolochia contorta* that grow in Korea were sampled under the permission of the local government (Table S5). Each species was identified based on the key morphological characters described previously [5]. The voucher specimens for all the four species were deposited in the herbarium of the National Institute of Biological Resources (KB), Incheon, Korea. Total genomic DNA was extracted using the DNeasy Plant Mini Kit (Qiagen Co., Hilden, Germany) following the manufacturer’s protocol. The purity of the extracted DNA was measured with a NanoDrop ND1000 (Thermo Fisher Scientific, Waltham, MA, USA), and the DNA was also checked visually through 1% agarose gel electrophoresis. After DNA fragmentation with an LE220-plus Focused-ultrasonicator (Covaris, Woburn, MA, USA), paired-end (PE) libraries were prepared using the TruSeq Nano DNA Kit (Illumina, San Diego, CA, USA) with a 670 bp average insert size. These libraries were sequenced on the Illumina MiSeq platform (Illumina Inc., San Diego, CA, USA), according to the manufacturer’s manual. To validate structural changes in the plastome of the section *Asiasarum*, ONT MinION long reads of *A*. *maculatum* were additionally generated. The Oxford Nanopore libraries were prepared in accordance with the manufacturer’s instructions, using a rapid sequencing kit SQK-RAD004 (Oxford Nanopore Technologies, Oxford, UK), with a 55 kb average insert size. The libraries were sequenced on the ONT MinION platform (Oxford Nanopore Technologies). All sequencing experiments were performed by PHYZEN Inc. (http://phyzen.com, South Korea).

### 4.2. Plastome Assembly and Annotation

For the plastome assembly, we followed the overall assembly pipeline of the dnaLCW method as described by Kim et al. [64]. In brief, high-quality trimmed reads with phred scores greater than 20 were obtained using the CLC quality trim tool, and then de novo assembly was conducted using the QIAGEN CLC Assembly Cell version 4.2.1 (QIAGEN Digital Insights, Aarhus, Denmark). The putative contigs of the plastome were selected and then mapped to reference sequences of *Asarum sieboldii* (MG551543) and *Aristolochia manshuriensis* (MN132862). Subsequently, the contigs were merged into a single draft sequence by connecting the overlapping terminal sequences. The draft plastome sequences were curated by remapping PE reads. The sequencing data of *A*. *maculatum* were obtained by ONT MinION base-calling using the Albacore version 2.0 (available online: https://github.com/Albacore/albacore) with default options. Trimming of the adapter and chimeric sequences was performed using Porechop (available online: https://github.com/rrwick/Porechop) with default options. Trimmed reads were *de novo* assembled using the SMARTdenovo program (available online: https://github.com/ruanjue/smartdenovo). A single contig was selected by comparing the plastome sequence assembled from Illumina MiSeq data. Gene annotation was performed using GeSeq (available online: http://chlorobox.mpimp-golm.mpg.de/geseq.html) [65], with tRNAscan-SE and BLAT search of default options. Circular maps were drawn using OGDRAW (available online: http://ogdraw.mpimp-golm.mpg.de/) [66]. Finally, the sequences of the plastomes of *Asarum* and *Aristolochia* species were deposited in GenBank (MN132858–MN132861).

### 4.3. Genome Structure and Comparative Analysis

We compared the overall genome structure, genome size, gene content, and repeats across all six Korean Aristolochiaceae species, and the previously reported plastomes of *A. sieboldii* (MG551543) and *Aristolochia manshuriensis* (MN132862) [43,67]. The GC content was compared using the Geneious Prime 2021.0.3 (Biomatters Ltd., Auckland, New Zealand). The Mauve version 2.4.0 software was used for aligning sequences and determining the rearrangements in the plastomes in Aristolochiaceae [68]. We also examined the sequence divergence among the six Korean Aristolochiaceae species through a sliding window analysis by computing π among the plastomes using the DnaSP version 6.0 [69]. For the sequence divergence analysis, we applied a window size of 600 bp with a 200 bp step size. Additionally, the whole plastomes of the four *Asarum* species were aligned using the MAFFT version 7.450 (available online: http://mafft.cbrc.jp/alignment/server/) and visualized using the Shuffle-LAGAN mode in mVISTA (http://genome.lbl.gov/vista/mvista/submit.shtml). For the mVISTA plot, we used the annotated plastome of *A. sieboldii* as a reference. Some misaligned regions were manually curated using BioEdit (available online: http://www.mbio.ncsu.edu/bioedit/bioedit.html). Thereafter, polymorphic regions showing SNPs and indels were investigated at the interspecific levels. 

We found repeat elements with two approaches. In the first approach, a web-based simple sequence repeats finder, MISA-web (available online: https://webblast.ipk-gatersleben.de/misa/), was employed to identify SSRs with thresholds of 10 repeat units for mono-, 5 repeat units for di-, 4 repeat units for tri-, and 3 repeat units for tetra-, penta-, and hexa-nucleotide SSRs. In the second approach, we investigated the size and type of LSRs in the plastome of Korean *Asarum* using Vmatch version 2.3.0 (available online: http://www.vmatch.de). The parameters were set as follows: a minimal repeat size of 40 bp, a minimal repetition number of two, and the type of LSRs (forward, reverse, palindromic, complementary).

### 4.4. Phylogenetic Analysis

Phylogenetic relationships were inferred using 26 species (31 accessions) in Aristolochiaceae as follows: four plastomes newly sequenced in this study (*A. heterotropoides*, *A*. *maculatum*, *A*. *misandrum*, *Aristolochia contorta*) and 27 previously reported CP genomes, including 16 *Asarum* species, 10 *Aristolochia* species, and *Saruma henryi* (Table S4). Two *Piper* species (*P*. *cenocladum* [DQ887677] and *P*. *kadsura* [KT223569]) were added as the outgroup. Due to structural variations and incomplete sequences in plastomes of this group, we used 79 protein-coding gene sequences. Protein-coding gene sequences were retrieved using annotation software Chloë (web application: https://chloe.plantenergy.edu.au/) and sorted alphabetically. After removing duplicated gene sequences, sorted sequences were concatenated and aligned using MAFFT and manually edited using the Geneious alignment viewer. Gaps in the sequences were treated as missing. We inferred the phylogeny using two approaches, the ML analysis and BI. We constructed the ML phylogeny using IQ-TREE version 1.6.11 with the TVM+F+R2 model [70] and 1000 bootstrap replicates for evaluating the node support. The nucleotide substitution model used in the phylogenetic analysis was chosen based on the Akaike information criteria implemented in the jModelTest version 2.1.10 [71]. BI phylogeny was reconstructed using MrBayes version 3.2.6 [72] under the following settings: a simple nucleotide model (lset nucmodel = 4by4, lset nst = 6), a proportion of invariable sites (lset rates = invgamma). The Markov chain Monte Carlo (MCMC) algorithm was applied for 5,000,000 generations after 25% burn-in, and sampling of trees every 1000 generations. The consensus trees were finally edited using the Figtree version 1.4.3 (available online: http://tree.bio.ed.ac.uk/software/figtree/).

In addition, we reassembled the plastome sequences of five *Asarum* species using *A. sieboldii* (MG551543) as a reference to better resolve the relationships among Korean *Asarum* species. Short reads generated by either Illumina HiSeq or the Ion Torrent Personal Genome Machine (PGM) were downloaded from NCBR Short Read Archive (SRA) and were assembled with Geneious Prime. The plastomes from six and three species belonging to section *Asiasarum* and *Heterotropa*, respectively, were aligned using MAFFT, and the AT-rich regions were excluded due to ambiguity. The ML phylogeny was constructed as stated above, except employing a model of TVM+F+I.

## Figures and Tables

**Table 1:** Plastome characteristics in the four Korean Aristolochiaceae species.

Scientific Name	*Asarum* *heterotropoides*	*Asarum* *maculatum*	*Asarum* *misandrum*	*Aristolochia* *contorta*
Genome length (bp)	190,168	193,105	193,163	160,556
LSC size (bp) (=SC in *Asarum*)	93,336	96,301	96,357	89,756
SSC size (bp)	-	-	-	19,882
Each IR size (bp)	48,416	48,402	48,403	25,459
Genomic structure	Tripartite ^(1)^	Tripartite ^(1)^	Tripartite ^(1)^	Quadripartite
GC contents (%)	36.78	36.24	36.22	38.49
No. of total genes	113	113	112	113
No. of protein-coding genes	79	79	78	79
No. of ribosomal RNA genes	4	4	4	4
No. of transfer RNA genes	30	30	30	30

^(1)^ functionally bipartite.

**Table 2:** List of genes within plastomes of three *Asarum* species and *Aristolochia contorta* in Korea.

Group of Genes	Three *Asarum* Species	*Aristolochia contorta*
Photosystem I	*psaA*, *psaB*, *psaC* (×2), *psaI*, *psaJ*, *ycf3* ^2^, *ycf4*	*psaA*, *psaB*, *psaC*, *psaI*, *psaJ*, *ycf3*^2^, *ycf4*
Photosystem II	*psbA*, *psbB*, *psbD*, *psbC*, *psbE*, *psbF*, *psbH*, *psbI*, *psbJ*, *psbK*, *psbL*, *psbM*, *psbN*, *psbT*, *psbZ*	*psbA*, *psbB*, *psbC*, *psbD*, *psbE*, *psbF*, *psbH*, *psbI*, *psbJ*, *psbK*, *psbL*, *psbM*, *psbN*, *psbT*, *psbZ*
Cytochrome b6/f	*petA*, *petB*^1^, *petD*^1^, *petG*, *petL*, *petN*	*petA*, *petB*^1^, *petD*^1^, *petG*, *petL*, *petN*
ATP synthase	*atpA*, *atpB*, *atpE*, *atpF*^1^, *atpH*, *atpI*	*atpA*, *atpB*, *atpE*, *atpF*^1^, *atpH*, *atpI*
Rubisco	*rbcL*	*rbcL*
NADH oxidoreductase	*ndhA*^1^ (×2), *ndhB* ^1^ (×2), *ndhC*, *ndhD* (×2), *ndhE* (×2), *ndhF* (×2), *ndhG* (×2), *ndhH* (×2), *ndhI* (×2), *ndhJ*, *ndhK*	*ndhA*^1^, *ndhB*^1^ (×2), *ndhC*, *ndhD*, *ndhE*, *ndhF*, *ndhG*, *ndhH*, *ndhI*, *ndhJ*, *ndhK*
Large subunit ribosomal proteins	*rpl2*^1^ (×2), *rpl14*, *rpl16* ^1^, *rpl20*, *rpl22* (×2), *rpl23* (×2), *rpl32* (×2), *rpl33*, *rpl36*	*rpl2*^1^ (×2), *rpl14*, *rpl16* ^1^, *rpl20*, *rpl22*, *rpl23* (×2), *rpl32*, *rpl33*, *rpl36*
Small subunit ribosomal proteins	*rps2*, *rps3* (×2), *rps4*, *rps7* (×2), *rps8*, *rps11*, *rps12* ^2^ (×2), *rps14*, *rps15* (×2), *rps16*^1^, *rps18*, *rps19* (×2)	*rps2*, *rps3*, *rps4*, *rps7*, *rps8*, *rps11*, *rps12*^2^ (×2), *rps14*, *rps15*, *rps16* ^1^, *rps18*, *rps19*
RNA polymerase	*rpoA*, *rpoB*, *rpoC1*^1^, *rpoC2*	*rpoA*, *rpoB*, *rpoC1*^1^, *rpoC2*
Unknown function genes	*ycf1* (×2), *ycf2* (×2)	*ycf1*, *ycf2* (×2)
Other genes	*accD*, *ccsA* (×2), *cemA* ^a^, *clpP* ^2^, *infA*, *matK*	*accD*, *ccsA*, *cemA*, *clpP*^2^, *infA*, *matK*
Ribosomal RNAs	*rrn16* (×2), *rrn23* (×2), *rrn4.5* (×2), *rrn5* (×2)	*rrn16* (×2), *rrn23* (×2), *rrn4.5* (×2), *rrn5* (×2)
Transfer RNAs	*trnA-UGC*^1^ (×2), *trnC-GCA*, *trnD-GUC*, *trnE-UUC*, *trnF-GAA*, *trnG-GCC*, *trnG-UCC* ^1^, *trnH-GUG*, *trnI-CAU* (×2), *trnI-GAU* ^1^ (×2), *trnK-UUU* ^1^, *trnL-CAA* (×2), *trnL-UAA* ^1^, *trnL-UAG* (×2), *trnM-CAU*, *trnfM-CAU*, *trnN-GUU* (×2), *trnP-UGG*, *trnQ-UUG*, *trnR-ACG* (×2), *trnR-UCU*, *trnS-GCU*, *trnS-GGA*, *trnS-UGA*, *trnT-GGU*, *trnT-UGU*, *trnV-GAC* (×2), *trnV-UAC* ^1^, *trnW-CCA*, *trnY-GUA*	*trnA-UGC*^1^ (×2), *trnC-GCA*, *trnD-GUC*, *trnE-UUC*, *trnF-GAA*, *trnG-GCC*, *trnG-UCC* ^1^, *trnH-GUG*, *trnI-CAU* (×2), *trnI-GAU* ^1^ (×2), *trnK-UUU* ^1^, *trnL-CAA* (×2), *trnL-UAA* ^1^, *trnL-UAG*, *trnM-CAU*, *trnfM-CAU*, *trnN-GUU* (×2), *trnP-UGG*, *trnQ-UUG*, *trnR-ACG* (×2), *trnR-UCU*, *trnS-GCU*, *trnS-GGA*, *trnS-UGA*, *trnT-GGU*, *trnT-UGU*, *trnV-GAC* (×2), *trnV-UAC* ^1^, *trnW-CCA*, *trnY-GUA*
No. of genes	113 (112 in *A. misandrum*)	113

^1^ Gene containing single intron; ^2^ Gene containing two introns; ^a^ Pseudogene in *A. misandrum*.

**Table 3:** The distribution and number of simple sequence repeats (SSRs) in four Korean *Asarum* species.

Unit Size								
Species	Mono	Di	Tri	Tetra	Penta	Hexa	C *	Total
*Asarum sieboldii*	76	20	15	6	547	241	134	1039
*A*. *misandrum*	77	20	15	6	536	244	139	1037
*A*. *maculatum*	78	18	12	6	536	243	135	1028
*A*. *heterotropoides*	77	17	10	7	494	232	116	953

* Compound SSR of which comprised more than two SSRs adjacent to each other.

## Data Availability

The four chloroplast genomes, newly sequenced in this study, were archived in NCBI with accession numbers (MN132858–MN132861).

## Supplementary Materials

The following are available online at https://www.mdpi.com/article/10.3390/plants10102056/s1, Figure S1: In silico validation of plastome junctions by Illumina MiSeq and Oxford Nanopore MinION read, Figure S2: Comparison of the plastome structure between fully and partially assembled plastomes of the same species, Figure S3: A ML phylogeny of *Asarum* species based on 186,718 bp of the assembled plastome with exclusion of the AT-rich regions, Figure S4: In silico validation of plastome junctions in the plastome sequences reassembled with sequences from NCBI SRA, Table S1: Sequencing and mapping results for newly sequenced five Korean Aristolochiaeae species, Table S2: Information of SSR across four Korean *Asarum* species, Table S3: Information of long sequence repeats across four Korean *Asarum* species, Table S4: Sequence information of species used in phylogenetic analysis, Table S5: Voucher information the Korean *Asarum* and *Aristolochia* species examined in this study.
