# Supplementary material for: Complete Plastome of Three Korean Asarum (Aristolochiaceae): Confirmation Tripartite Structure within Korean Asarum and Comparative Analyses
Source: Plants (Basel). 2021 Sep 29;10(10):2056. doi: 10.3390/plants10102056 (PMC8540983; doi:10.3390/plants10102056)
Supplement: Supplementary file 1 [file plants-10-02056-s001.zip › plants-1389948-supplementary- Figures S1-S4.pdf]

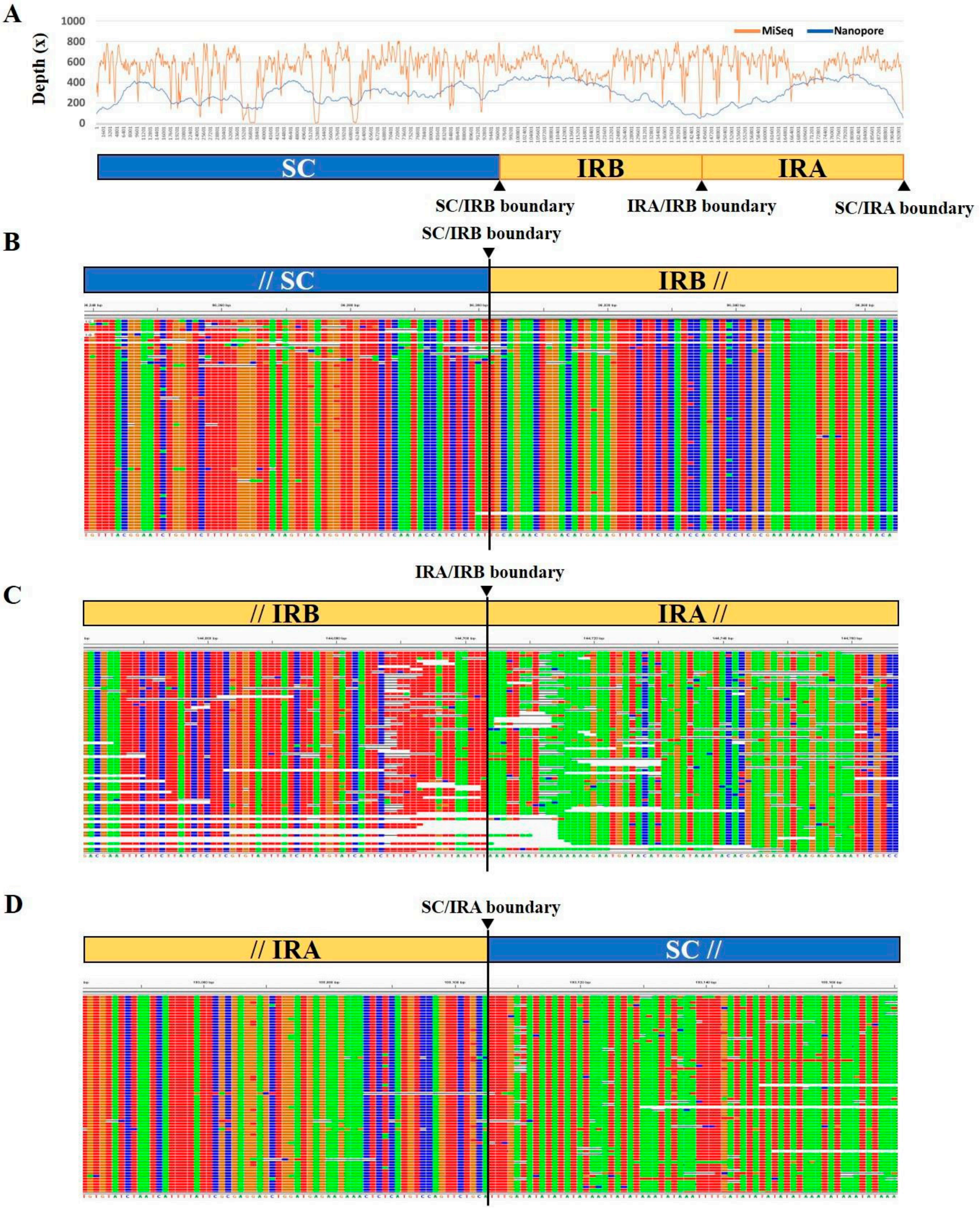

Figure S1. *In silico* validation of chloroplast genome junctions by Illumina MiSeq and Oxford Nanopore MinION read. (**A**) The depth distribution of Illumina and Nanopore read. The orange and blue lines indicate the depth of the Illumina and Oxford Nanopore reads, respectively. The schematic diagram under the graph indicates the tripartite CP genome structure of *Asarum maculatum*. (**B**) The mapped read-status of SC/IRB boundary. (**C**) The mapped read-status of IRA/IRB boundary. (**D**) The mapped read-status of SC/IRA boundary.

(A) **Korean *Asarum sieboldii* plastome (MG551543; 193,356 bp)**

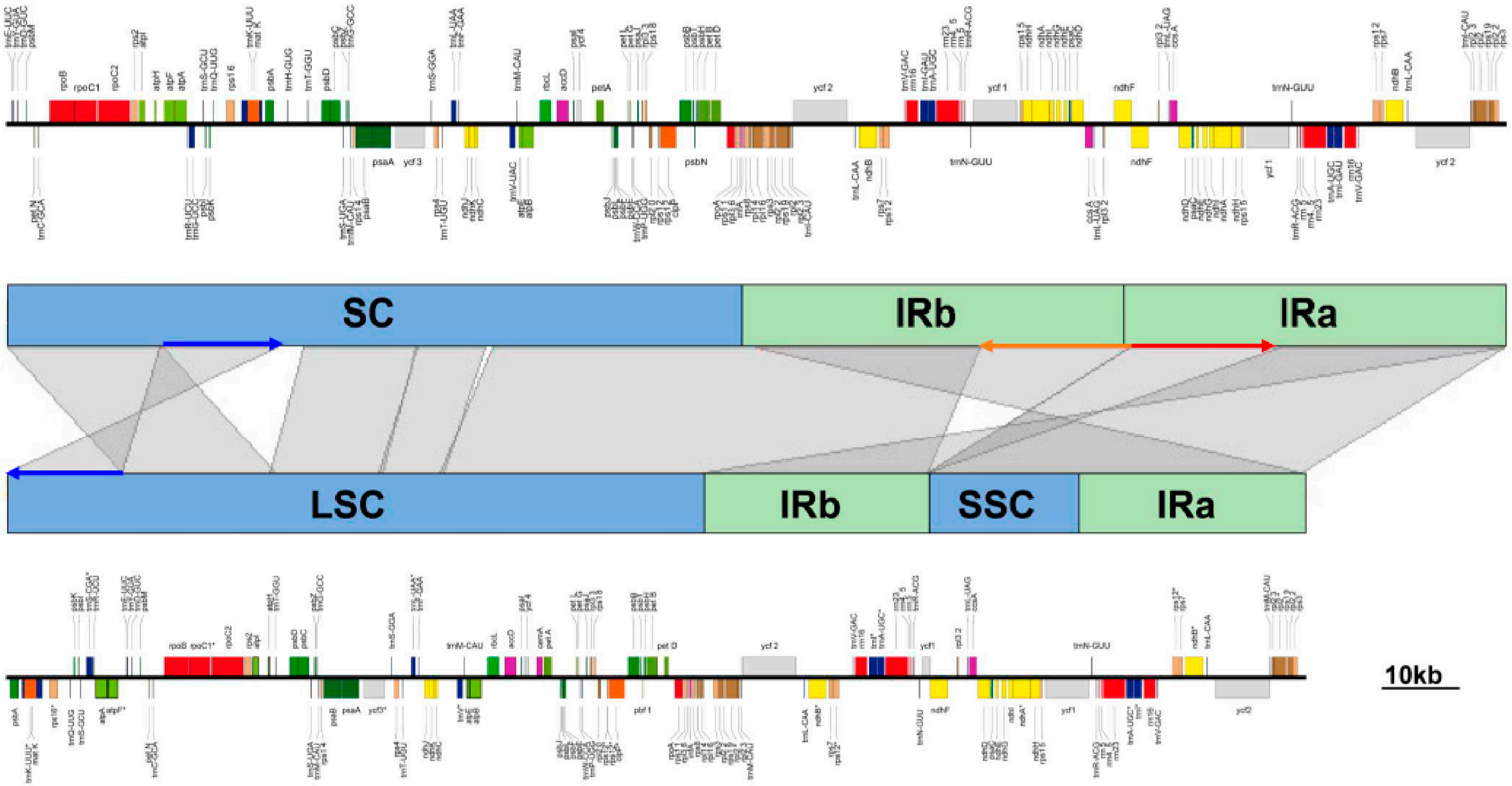

Chinese *Asarum sieboldii* plastome (MW034667; 167,293 bp)

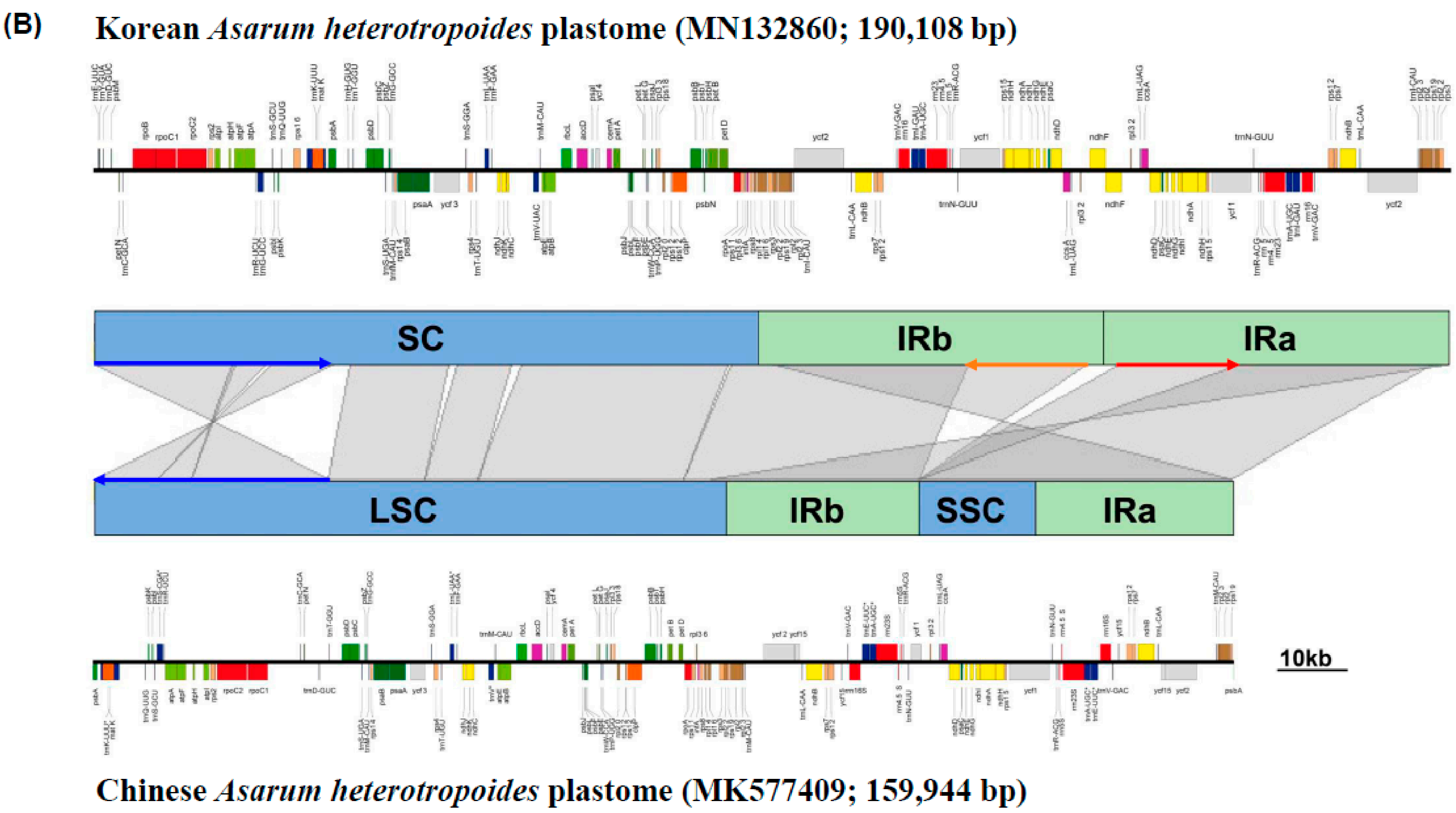

Figure S2. Comparison of the plastome structure between fully and partially assembled plastomes of the same species. (**A**) *A. sieboldii*. (**B**) *A. heterotropoides*

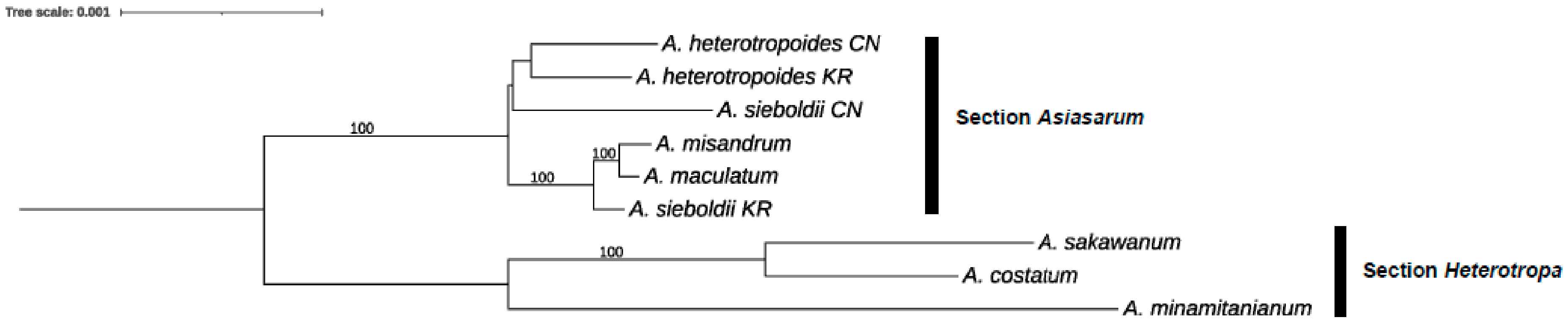

Figure S3. A ML phylogeny of Asarum species based on 186,718 bp of the assembled plastome with exclusion of the AT-rich regions. The number on each node indicate the bootstrap value (> 50 %). CN and KR next to taxon name indicate Chinese and Korean accession, respectively. Sequences downloaded from NCBI Sequence Read Archive (SRA) were mapped onto the plastome of *A. sieboldii* (MG551543) using Geneious Prime 2021.0.3: *A. heterotropoides* CN (SRR14433893), *A. sieboldii* CN (SRR13221845), *A. costatum* (DRR124157), *A. minamitanianum* (DRR124158), *A. sakawanum* (DRR075510).

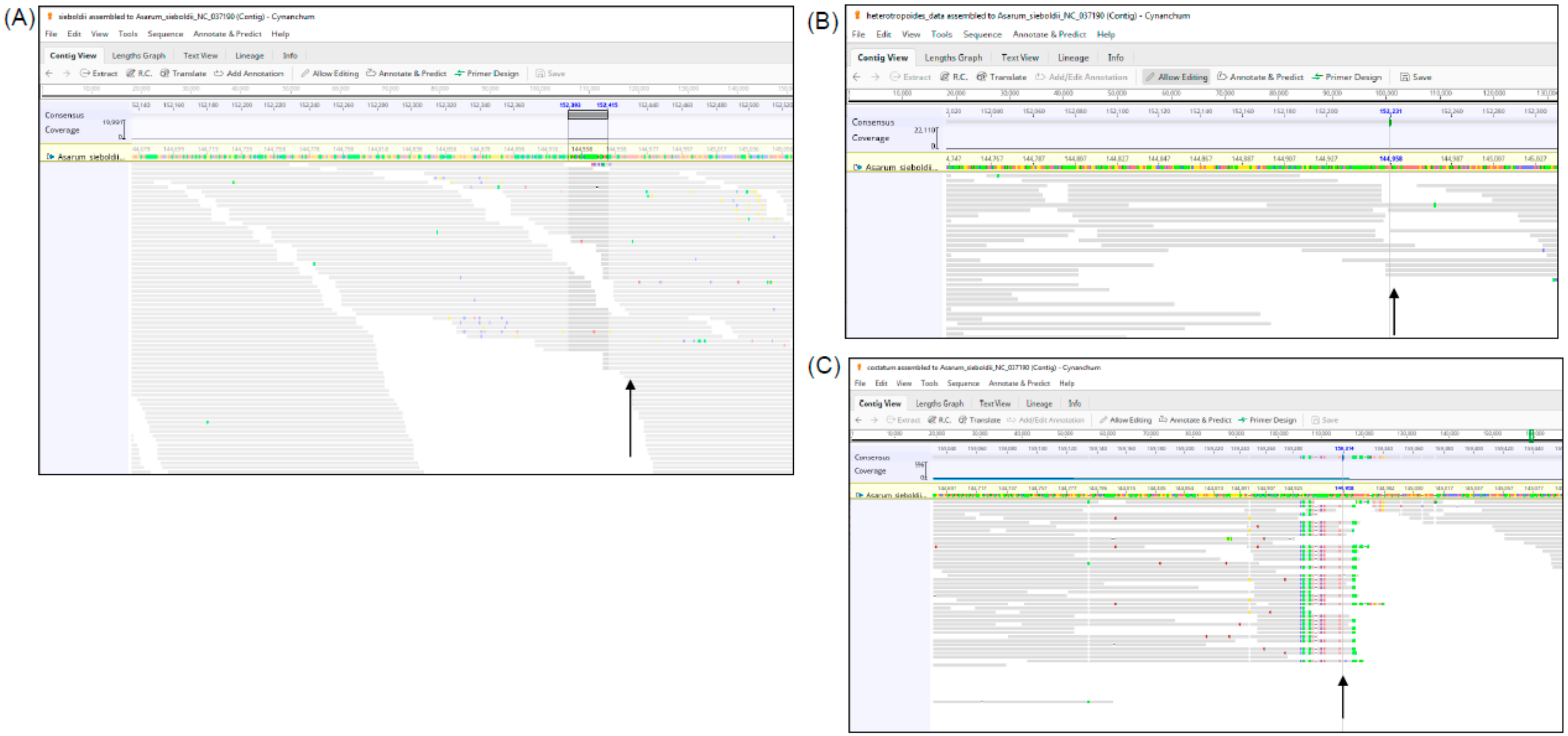

Figure S4. *In silico* validation of plastome junctions in the plastome sequences reassembled using *A. sieboldii*(MG551543) as a reference using sequences from NCBI SRA. Arrow indicates the IRA/IRB boundary. (**A**) *A. sieboldii* (SRR13221845). (**B**) *A. heterotropoides* (SRR14433893). (**C**) *A. costatum*(DRR124157).
